# Mutagenicity Assessment to Pesticide Adjuvants of Toluene, Chloroform, and Trichloroethylene by Ames Test

**DOI:** 10.3390/ijerph18158095

**Published:** 2021-07-30

**Authors:** Jing Zhang, Wenqiang Wang, Zhoutao Pei, Jingya Wu, Ran Yu, Yimin Zhang, Liwei Sun, Yuexiang Gao

**Affiliations:** 1 School of Energy & Environment, Southeast University, Nanjing 210096, China; 220190584@seu.edu.cn (J.Z.); 220190511@seu.edu.cn (W.W.); 220170623@seu.edu.cn (Z.P.); 220200551@seu.edu.cn (J.W.); yuran@seu.edu.cn (R.Y.); 2 Taihu Lake Water Environment Engineering Research Center (Wuxi), Southeast University, Wuxi 214061, China; 3 Nanjing Institute of Environmental Sciences, Ministry of Ecology and Environment of the People’s Republic of China, Nanjing 210042, China; zym@nies.org

**Keywords:** toluene, chloroform, trichloroethylene, mutagenicity, Ames test, PAs, pesticides

## Abstract

Pesticide adjuvants (PAs) denote the general term for auxiliaries in pesticide preparations except for the active components. Toluene, chloroform, and trichloroethylene are the three most commonly used PAs as organic solvents. The residues of the three chemicals in the process of production and application of pesticides may endanger the ecosystem. In the present study, the mutagenicity of toluene, chloroform, and trichloroethylene as well the mixture of the three chemicals was tested by the *Salmonella typhimurium* reverse mutation test (Ames test) with TA97, TA98, TA100, and TA102 strains in the system with and without rat liver microsomal preparations (S9). The four tester strains have been used for more than 40 years to detect mutagenic compounds in chemicals, cosmetics, and environmental samples. The mutagenicity was detected on tester strains in the separated experiment from the three chemicals. The addition of S9 decreased the mutation ratios of toluene to four strains, except for the TA100 strain, but increased the mutation ratios of chloroform to four strains except for the TA98 strain. Trichloroethylene caused positive mutagenicity to become negative on the TA102 strain. In the mixed experiment, positive effects were detected only on the TA102 strain in the absence of S9. The addition of S9 increased the mutagenicity except for the TA102 strain. The mixture of toluene, chloroform, and trichloroethylene showed antagonism in mutagenicity to tester strains, except for the TA102 strain without S9. However, the mixture showed a synergistic effect to tester strains after adding S9 except for the TA98 strain.

## 1. Introduction

Pesticide adjuvants (PAs) are all auxiliaries in pesticide formulation except for the active components [1]. The addition of PAs can increase the biological activity of the active ingredients or make the formulation chemically more stable [2]. The content of PAs in pesticides is up to 99.9% [3]. In pesticide application, most PAs are accompanied by spraying into the soil and are subsequently washed directly into the water body [4]. Some highly volatile PAs directly volatilize into the air, resulting in organic pollutants in the atmosphere [5,6]. Studies have shown that more than 80% of PAs residues remain in the soil, water, and atmosphere environmental media during pesticide application [7].

PAs were usually regarded as inert ingredients in pesticides [8]. Most of the tests to meet the requirements of registering pesticides are related to the active ingredients only, exclusive of the PAs. However, most PAs were reported to be hazardous and cause risks to environmental organisms and human health. According to the study by the EPA, more than 1000 PAs were at medium toxicity, and some PAs exhibited carcinogenicity, teratogenicity, and endocrine disruption [9]. Various adjuvants were detected in fruits and vegetables [10,11]. Moreover, fourteen PAs were proven to have a growth-inhibition effect on *Scenedesmus quadricauda* and *Chlorella vulgaris* [12]. In addition, PAs were proven to increase the ecotoxicity of pesticide formulations in nontarget plants, animals, and microorganisms [13,14,15,16]. However, few studies focus on the residue levels, environmental fate, and toxicity of PAs. Indeed, toxicity data are still lacking. Therefore, regarding toxicity, there is an urgent need to assess environmental and human health risks presented by PAs [17].

Based on the investigation of a pesticide-contaminated site, toluene, chloroform, and trichloroethylene were the three most frequently detected PAs. Toluene, chloroform, and trichloroethylene were used as organic solvents of the active ingredient, consistently detected in the natural soil environment. For example, toluene was detected in an agricultural area from Shenyang at 0.15 mg/kg [18]. The highest concentration of chloroform in a contaminated site in southern China is 38.1mg/g [19]. Toluene, chloroform, and trichloroethylene were all detected in a pesticide-contaminated site in Changzhou [20].

There were a few ecotoxicological studies on toluene, chloroform, and trichloroethylene. Ya-Wei Fan studied the aquatic ecotoxicological effects of toluene on zebrafish (*Brachydanio rerio*), *Daphnia* *magna*, and *Limnodrilus hoffmeisteri*, showing medium toxicity, low toxicity, and low toxicity to the three organisms, respectively [21]. The 50% lethal concentration (LC_50_) of toluene to *Drosophila melanogaster* was 430 mg/L [22]. The 50% growth inhabitation (EC_50_) for toluene on sulfur-oxidizing bacterial (SOB) was 1.2 mg/kg [23].The trichloroethylene significantly inhibited the growth of cyanobacterium strain (*Synechococcus* PCC 6301) [24]. To assess genotoxicities and effects on reproduction, studies conducted report that toluene can cause chromosomal aberration in animal embryos, chloroform exhibited reproductive and developmental toxicity, and trichloroethylene showed teratogenic effects on animal hearts [25,26].

However, there are few reports on the mutagenicity of toluene, chloroform, and trichloroethylene. The *Salmonella typhimurium* reverse mutation test (Ames test) is a worldwide used method according to the Hygienic Standards for Cosmetics and the OECD guidelines [27,28]. The method employs histidine auxotrophic-deficient *Salmonella* strains to detect the mutagenicity of chemicals. The Ames test can also detect the mixture mutagenicity quickly and accurately. Some research successfully reports the separated and mixed mutagenicity of Benzophenones (BPs) using the Ames test [29].

In this study, the separated and mixed mutagenicity of toluene, chloroform, and trichloroethylene were evaluated by the Ames test to study its mutagenic effects. The results of this study will improve the genotoxicity database of PAs and provide a more scientific basis for the future ecological risk management of PAs.

## 2. Materials and Methods

### 2.1. Origin of Chemicals

The toluene (CAS: 108-88-3; purity > 95%), chloroform (CAS: 67-66-3; purity > 95%), and trichloroethylene (CAS: 79-01-6; purity > 95%) were purchased from Aladdin Biochemical Technology Co., Ltd., (Shanghai, China). Dimethyl sulfoxide (CAS: DMSO) (67-68-5; purity > 99.5%) was obtained from Sinopharm Chemical Reagent Co., Ltd., (Shanghai, China), which was used as a solvent. Liver microsomal S9 was purchased from CHI Scientific, Inc., (Wuxi, China).

### 2.2. Test Stains

Four histidines auxotrophic *Salmonella typhimurium* strains, namely TA97, TA98, TA100, and TA102, strains were obtained from Jiangsu Provincial Center for Disease Control and Prevention and Guizhou Medical University. These were stored in an ultra-low temperature refrigerator at −80 °C. The genotypes of the strains should be confirmed after receiving the cultures.

In each experiment, the cultures were incubated in nutrient broth for 10 h at 37 °C with shaking until the concentration of bacteria was 1–2 × 10^9^ cells/mL.

### 2.3. Salmonella Typhimurium Reverse Mutation Test (Ames Test)

The experiment was performed according to the OECD testing guideline [28] and Safety and Technical Standards for Cosmetics [27]. The test was carried out with or without liver microsomal metabolic activation (S9) mixture, prepared by standard method. Five dose groups (0.5, 2.5, 5, 25, 50 μL/plate) were carried out for testing the chemicals.

During the experiment, the top layer medium was kept warm in a 45 °C water bath. Then, each glass tube was successively supplemented with 0.1 mL of the *S. typhimurium* strains, 0.1 mL of the tester chemical, 0.5 mL of S9 mixture (when metabolic activation is required), and 2.0 mL of the top layer medium. The mixture was thoroughly mixed with a vortex mixer and quickly poured into the bottom plate. The plate was shaken to distribute the top layer evenly. All the plates for the experiments were incubated under the same conditions: 37 ± 0.5 °C, 48 h, dark.

Negative control (blank and solvent) experiment was conducted to determine the quality of the strains and whether the solvent DMSO can cause mutagenicity on the tested strains. In the blank control, plates were poured with LB medium and 0.1 mL bacterial. In the solvent DMSO control, 0.1 mL bacterial and 0.1 mL DMSO, the highest dose, was added to the medium. The culture conditions were the same as the test compounds group. Each dose of tested compounds and the negative control (blank and solvent) groups were performed in triplicate.

The mixed Ames test was designed based on the results in the separated experiment. The chemical was mixed at a 1:1:1 ratio according to the highest effective concentration between the four tester strains in the separated experiment without S9. The highest concentration with a positive result of toluene, chloroform, and trichloroethylene was 50 μL/plate, 10 μL/plate, and 10 μL/plate, respectively, recorded as the initial concentration 100%. Then, the mixture was diluted in sequence according to the concentration gradient of 100%, 50%, 10.0%, 1.0% and 0.1%.

Testing the toluene, chloroform, and trichloroethylene mixture was conducted with the same method as for the separated compounds.

### 2.4. Statistical Analysis

The mutagenicity ratio (MR) is calculated as

(1) $$\mathrm{MR}=X/X_{0}$$

where *X* is the number of reverse mutation colonies on the chemical-treated plates, and *X*_0_ is the average number in the DMSO and blank control group. When the mutagenicity ratio (MR) is ≥2, and the background is normal, the mutagenesis is positive. All values are expressed as mean ± SD. Statistical analysis was performed with the Origin software (ver.2020). *p* < 0.05 was considered statistically significant.

## 3. Results

### 3.1. Negative Control Results

In all the tests, the number of reverse mutation colonies in the blank and DMSO control was between 90 and 180 for TA97 strain, 30–50 for TA98 strain, 100–200 for TA100 strain, and 240–320 for TA102 strain, which met the quality control requirements for tester strains in the Ames test [30,31]. The results also proved that the DMSO dose used in this study did not cause mutagenicity.

### 3.2. Ames Test for Toluene

**Figure 1:**
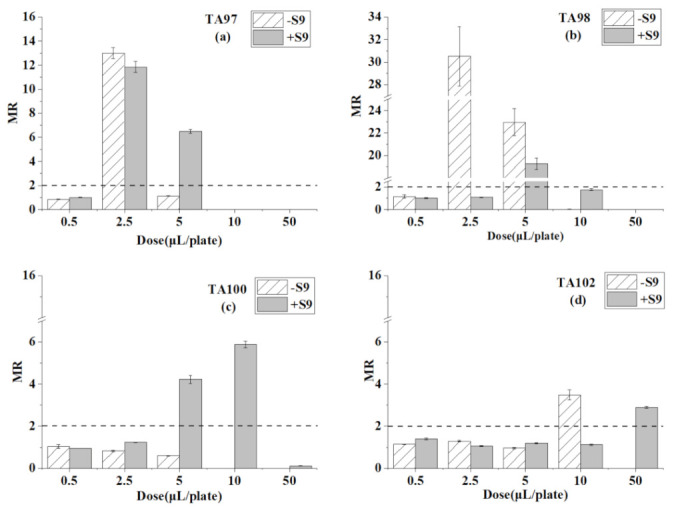
Mutagenesis of toluene to four strains in the presence and absence of S9 liver extract; (**a**) TA97 strain; (**b**) TA98 strain; (**c**) TA100 strain; (**d**) TA102 strain. The mutagenicity ratio (MR) is the average ratio (±SE) from three parallel experiments.

Table 1 presents the reverse mutation colonies of four *S. typhimurium* strains after exposure at different doses of toluene with and without S9 in the Ames test. The MR values of toluene at every dose are compared in Figure 1.

For the TA97 strain (Table 1 and Figure 1), mutagenicity was detected only at the dose of 2.5 μL/plate. After adding S9, mutagenicity was detected at two doses, 2.5 and 5 μL/plate. When the concentrations were higher than 10 μL/plate, there was no colony growth on the plate both with and without S9. For the TA98 strain, the result was in contrast to the TA97 strain, mutagenicity appeared at the dose of 2.5 μL/plate and 5 μL/plate without S9, while in the system with S9, positive results only appeared with 5 μL/plate. The concentrations higher than 10 μL/plate of toluene showed strong inhabitation on TA98 strain growth. For the TA100 strain, the mutagenicity did not appear at any dose in the absence of S9. However, after the metabolic activity of S9, the mutation rate increased significantly, the mutagenicity was detected at 5 μL/plate and 10 μL/plate. Similarly, the highest concentrations of toluene at 50 μL/plate had an inhibitory effect on the TA100 strain. For the TA102, toluene showed mutagenicity at the dose of 10 μL/plate without S9 and the positive results appeared at the highest dose, 50 μL/plate with S9.

In conclusion, toluene caused mutagenicity on the TA97, TA98, and TA102 strains without S9. After adding S9, toluene further caused mutagenicity on the TA100 strain. Therefore, mutagenicity was detected on all tester strains. The higher concentrations inhibited tester strains, especially for the TA 97 and TA 98 strains.

### 3.3. Ames Test for Chloroform

**Figure 2:**
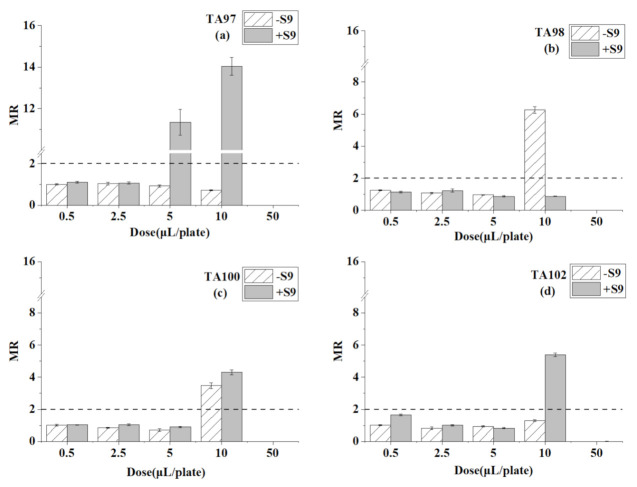
Mutagenesis of chloroform to four strains in the presence and absence of S9 liver extract; (**a**) TA97 strain; (**b**) TA98 strain; (**c**) TA100 strain; (**d**) TA102 strain. The mutagenicity ratio (MR) is the average ratio (±SE) from three parallel experiments.

Table 2 presents the reverse mutation colonies of four *S. typhimurium* strains at different doses of chloroform with or without S9 in the Ames test. The MR values of chloroform are compared in Figure 2.

**Figure 3:**
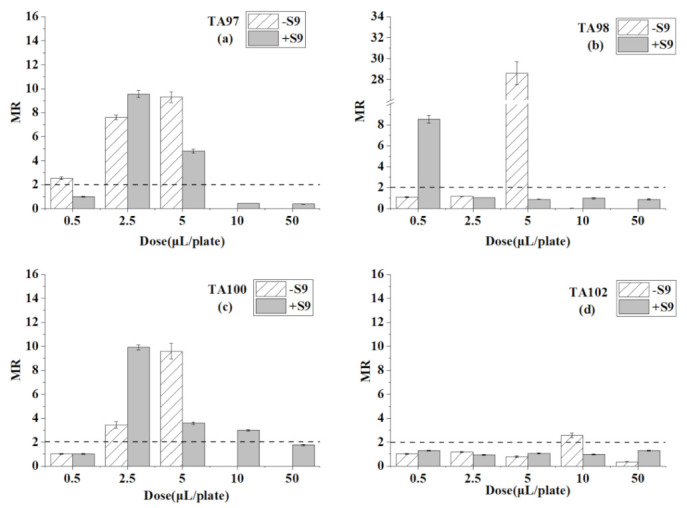
Mutagenesis of trichloroethylene to four strains in the presence and absence of S9 liver extract; (**a**) TA97 strain; (**b**) TA98 strain; (**c**) TA100 strain; (**d**) TA102 strain. The mutagenicity ratio (MR) is the average ratio (±SE) from three parallel experiments.

For the TA97 strain (Table 3 and Figure 3), in the system without S9, chloroform had no mutagenicity at any dose. When the S9 was added, mutagenicity was detected at the dose of 5 and 10 μL/plate. For the TA98 strain, chloroform caused a positive effect only at the dose of 10 μL/plate without S9. After S9 metabolism, the mutagenicity disappeared at all designed doses. For the TA100 strain, in the system with or without S9, mutagenicity was both detected only at the dose of 10μL/plate, the mutation rate increased after S9 metabolism. As for the TA102 strain, the results were negative at all doses. After adding S9, mutagenicity was detected at the dose of 10 μL/plate. 

Briefly, chloroform caused positive mutagenicity to the TA98 and TA100 strains without S9. After adding S9, mutagenicity was detected on the TA97, TA98, and TA102 strains. No colonies were observed at the highest 50 μL/plate dose both with or without S9, which showed an inhibitory effect on tester strains by a high concentration of chloroform.

### 3.4. Ames Test for Trichloroethylene

Table 3 presents the reverse mutation colonies of four *S. typhimurium* strains at different doses of trichloroethylene with and without S9 in the Ames test. The MR value of trichloroethylene at every dose is compared in Figure 3.

For the TA97 strain (Table 3 and Figure 3), mutagenicity was detected at the dose of 0.5–5 μL/plate, and the mutation rate increased with the dose. After S9 metabolism, the mutation ratios decreased but also appeared positive at 2.5 and 5 μL/plate. When the dose was higher than 10 μL/plate, the colonies were not observed, showing an inhabitation effect on the TA97 strain. For the TA98 strain, significant mutagenicity was detected at the dose of 5 μL/plate without S9. However, the colonies numbers sharply down to near zero when the concentrations were higher than 5 μL/plate. After adding S9, mutagenicity appeared at the lowest dose, 0.5 μL/plate, and increased the mutagenic ability of trichloroethylene. For the TA100 strain, trichloroethylene caused mutagenicity at 2.5 and 5 μL/plate, and the results were as same as the TA98 strain without S9. With the addition of S9, the mutagenicity was detected at the three doses, from 2.5 to 10 μL/plate. As for the TA102 strain, positive mutagenicity appeared only at the dose of 10 μL/plate, and the mutagenicity disappeared after the addition of S9.

From the results of the four strains, trichloroethylene caused positive mutagenicity to all tester strains without S9, and to the TA97, TA98, and TA100 strains with S9. The concentration greater than 10 μL/plate of trichloroethylene caused inhabitation on the TA97, TA98, and TA100 strains in the absence of S9.

### 3.5. Ames Test for the Mixtures of Toluene, Chloroform, and Trichloroethylene

**Figure 4:**
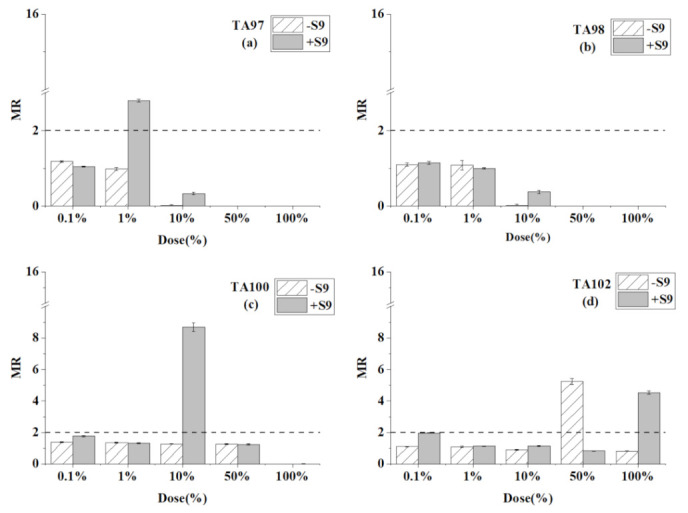
Mutagenesis of the mixtures of toluene, chloroform, and trichloroethylene to four strains in the presence and absence of S9 liver extract; (**a**) TA97 strain; (**b**) TA98 strain; (**c**) TA100 strain; (**d**) TA102 strain. The mutagenicity ratio (MR) is the average ratio (±SE) from three parallel experiments.

Table 4 presents the reverse mutation colonies of four *S. typhimurium* strains at different doses of the mixture of toluene, chloroform, and trichloroethylene with and without S9. The MR values at every dose are compared in Figure 4.

For the TA97 strain and TA98 strain without S9 (Table 4 and Figure 4), no mutagenicity was detected at any ratio. Moreover, when the ratios were greater than 10%, no colony growth was observed. The only difference is that the mixture caused mutagenicity to TA97 strain at 1% ratio with S9. For the TA100 strain, the positive result was only detected at 10% with the addition of S9. The mixture showed an inhibiting effect on the TA100 strain at the highest ratio. For the TA102 strain, mutagenicity was only detected at 50% without S9, and at 100% ratio by adding S9.

In a word, the mixture of toluene, chloroform, and trichloroethylene caused positive mutagenicity only on the TA102 strain without S9. After S9 addition, the mixture caused positive mutagenicity to the TA97, TA100, and TA102 strains. Furthermore, no mutation colonies were observed at a higher dose ratio on the TA 97, TA98, and TA100 strains.

## 4. Discussion

### 4.1. Mutagenicity Analysis

Considering the results, toluene caused mutagenicity on TA97, TA98, and TA102 strains, chloroform caused mutagenicity on TA98 and TA100 strains, and trichloroethylene caused mutagenicity on all four tester strains without S9.

There were few studies about the mutagenicity of toluene on *Salmonella typhimurium*. However, a study showed that toluene is responsible for higher genotoxicity in rotogravure printers [32]. Chloroform was reported about its mutagenic effect on *Salmonella typhimurium* TA98 and TA100 strains without S9, which is consistent with the results of this study [33]. Although some studies reported the non-mutagenic nature of trichloroethylene [34], the dose designed was much lower than that in this study, and Harrington-Brock concluded that a high dose of trichloroethylene can cause mutagenicity [35]. Our results indicated that toluene, chloroform, and trichloroethylene and the mixture all showed significant mutagenicity on the four tester strains both in the systems with and without S9. However, the results did not show the regular dose effects. The work of Balasubramanyam and Wen-Qian Wang also showed a similar phenomenon [29,36].

According to the Ames test principle, TA97 and TA98 strains detect various frameshift mutagens while TA100 and TA102 strains detect mutagens that cause base-pair substitutions [30]. Therefore, toluene, chloroform, and trichloroethylene caused both various frameshift mutagens and base substitution mutations, while the mixtures of the three chemicals only caused base substitution mutations.

According to our results, the lowest mutagenic concentration of toluene, chloroform, and trichloroethylene to the tester strains was 2.5, 10, and 0.5 μL/plate, respectively. The corresponding effective dose of trichloroethylene is the lowest, followed by toluene and chloroform. Therefore, the most effective mutagenic is trichloroethylene, the weakest is chloroform, and toluene is the middle. A study regarding the correlation between the toxicity and structure of hall hydrocarbons proved that the longer the carbon chain, the stronger the toxicity when the number of halogens is fixed [37]. This may explain why the toxicity of chloroform was much lower than trichloroethylene. Although few studies have been undertaken comparing the toxicity of chloroform with that of BTEX (Monoaromatic Hydrocarbons),the toluene and chloroform showed 99% and 40% mortality in 48-h acute toxicity tests using the freshwater invertebrate *Ceriodaphnia dubia,* respectively [38]. Other studies also indicated chloroform’s toxicity is lower than toluene’s toxicity according to quantitative structure–activity relationship (QSAR) analysis [39,40]. Our mutagenicity result was consistent with these toxicity research reports.

### 4.2. Mutagenicity Change by S9 Liver Particles Metabolism

S9 was used to detect the mutagenicity changes of toluene, chloroform, and trichloroethylene after in vitro metabolism, which partly reflect the mutagenicity performance of the three chemicals in the actual environment. Generally, the mutation ratio results in the system with or without S9 were different due to the metabolic activity of S9 liver particles.

For toluene, the effective doses that caused mutagenicity were higher in the S9 added systems than those without S9 for four strains. Furthermore, for the TA100 and TA102 strains, mutagenicity was detected at the doses that caused an inhibition effect on the strains without S9. These results indicated that the mutagenicity of toluene at a higher dose may be activated by the S9 metabolism, as its chemical properties were changed, and therefore caused mutagenicity.

For chloroform, the mutagenicity was changed from negative to positive on TA97 and TA102 strains, and this result is similar to that of toluene, where the mutagenicity was activated by the S9 metabolism. The mutagenicity type detected by TA97 and TA102 strains was different, and thus the metabolism by S9 increased the possibility of mutagenicity in four strains. This result indicated that the risk of the metabolite from chloroform is higher than the original chemical itself, so the behavior of the chemical in the received environment needed to be monitored with vigilance.

For trichloroethylene, the mutagenicity changes were related to the experimental dose for the TA98 and TA100 strains, which caused mutagenicity at a lower dose and negative dose without S9. A study has also proven the mutagenicity of trichloroethylene metabolites by the Ames test [41]. Similar to toluene and chloroform, S9 activated the mutagenicity of trichloroethylene.

For the mixture of three chemicals, i.e., toluene, chloroform, and trichloroethylene, the mutagenicity changed from negative to positive for TA97 and TA100 strains, and the mutagenicity type was changed with S9.

Generally, the mutagenicity was activated by the S9 metabolism for the three chemicals and their mixture. However, the mutagenicity changes were complex, depending on the tester strains and the experimental dose. The Ames test results cannot explain the actual metabolic mechanism and its behavior in the environments. The toluene, chloroform, and trichloroethylene could be metabolized by numerous microorganisms in the actual environment. Therefore, the mutagenicity changes were more complex than the laboratory results. However, it must be pointed out that the three chemicals and the mixture have the potential risk of increased mutagenicity after metabolism in the environment according to our study results.

### 4.3. The Mixed Test Results

The toluene, chloroform, and trichloroethylene aare usually present in the actual environment in mixed rather than in separated conditions. Joint toxicity data are lacking due to the complex reaction. The mixed experiment results of the three chemicals in the study were also different from those in the separated experiments.

In the system without S9, for the TA97, TA98, and TA100 strains, the toluene, chloroform, and trichloroethylene mixture showed no mutagenicity at any dose ratio, while positive results were detected in the separate experiments at the corresponding dose. The mutagenicity in the mixture was reduced for TA97, TA98, and TA100 strains. For the TA102 strain, mutagenicity was detected at 50% of the mixture, in which the concentration of toluene, chloroform, and trichloroethylene was 25 μL/plate, 5 μL/plate, and 5 μL/plate. However, in the separated experiments for TA102 strain, chloroform and trichloroethylene both show negative results at the dose of 5 μL/plate. Toluene caused mutagenicity at the dose of 10μL/plate but shown inhibit effect at the dose of 50 μL/plate, so at the dose of 25 μL/plate, the mutagenicity result was unsure. There were two possible speculations of mutagenicity in the mixture for TA102 strain. First, the mutagenicity shown in the mixture was from toluene when toluene caused mutagenicity at 25 μL/plate. Second, the mutagenicity after mixing is increased, and a synergistic effect was exhibited when toluene showed a negative result at 25 μL/plate.

After S9 metabolism, the results were different from the -S9 system. In the mixed test for the TA97 strain, mutagenicity was observed at 1%, in which the concentration of toluene, chloroform, and trichloroethylene was 0.5 μL/plate, 0.1 μL/plate, and 0.1 μL/plate, respectively. However, the lowest corresponding dose caused mutagenicity of toluene, chloroform, and trichloroethylene was 2.5 μL/plate, 5 μL/plate, and 2.5 μL/plate, respectively, in the separate experiment. The results prove that the dose that caused positive mutagenicity of the three chemicals in the mixture was lower than that in the separated test, indicating that the mixing of toluene, chloroform, and trichloroethylene to TA97 strain increased their mutagenicity. For the TA100 strain, the result was similar to the TA97 strain, as the lowest corresponding dose in the mixed teat was lower than the separate results, indicating a synergistic effect. For the TA102 strain, the effective dose of toluene and chloroform in the mixed test was the same as that in the separate test. However, the mixed effect dose of trichloroethylene was lower than the separate effect dose, showing a synergistic effect to TA102 strain.

In a word, the mixture presented an antagonistic effect to tester strains except for the TA102 strain without S9. Studies have proven that the mixture of BP and BP-1 caused antagonistic mutagenicity, similar to our results [29]. However, the mixture showed a synergistic effect for TA97, TA100, and TA102 strains with S9. The results indicate the toxicity complexity of mixed chemicals in the actual environment. At present, few researchers reported the mechanism of mixing toxicity. Studies have shown that the mixed chemicals may lead to different eco-toxic effects on different organisms [42,43]. The results proved that the mixture in the actual environment had a potential increased mutagenicity risk.

In practice, the mixture of PAs, not only a simple mixture of adjuvants, is usually used in combination with pesticide ingredients. Their interaction is potentially crucial to overall efficacy and the toxicity performance will be more complicated. The results of this study partly support this complexity and provided a scientific basis for evaluating the ecological risk of PAs and their mixtures. Future studies should be conducted on more biota species and biological levels with respect to PAs. At the same time, the regulations governing pesticides need to be improved by adding system provisions concerning the dosage and usage of PAs.

## 5. Conclusions

(1) In the separated test, toluene, chloroform, and trichloroethylene caused mutagenicity to the tester strains in the system without S9. The addition of S9 decreased the mutagenicity of toluene to four strains except for the TA100 strain, but increased the mutagenicity of chloroform to four strains except for the TA98 strain. Trichloroethylene changed the positive mutagenicity to negative on TA102 strain. The mutagenicity of the three chemicals was activated by the S9 metabolism.

(2) The mixture of toluene, chloroform, and trichloroethylene caused positive mutagenicity only on TA102 strain without S9. The addition of S9 further caused mutagenicity on TA97 and TA98 strains. The mixture of the three chemicals exhibited different mutagenicity in comparison to the separated experiment.

(3) The mixture of toluene, chloroform, and trichloroethylene showed antagonism in mutagenicity to tester strains except for TA102 strain without S9, while the mixture showed a synergistic effect except for TA98 strain after adding S9. The mechanism of the mixing effects and the change of mutagenicity after S9 metabolism require further study.

## Figures and Tables

**Table 1:** Reverse mutation colonies of four *S. typhimurium* strains exposed to toluene with and without S9.

Chemicals	Dose (μL/plate)	TA97		TA98		TA100		TA102	
		-S9	+S9	-S9	+S9	-S9	+S9	-S9	+S9
DMSO	100	109 ± 5	95 ± 3	31 ± 1	37 ± 1	185 ± 6	171 ± 6	256 ± 12	254 ± 5
	0	107 ± 7	93 ± 3	33 ± 1	36 ± 2	191 ± 9	165 ± 10	221 ± 11	250 ± 5
toluene	0.5	92 ± 4	94 ± 3	37 ± 4	37 ± 2	195 ± 17	159 ± 1	273 ± 2	355 ± 11
	2.5	1400 ± 50 *	1112 ± 43 *	981 ± 84 *	39 ± 1	155 ± 7	207 ± 2	308 ± 8	266 ± 7
	5	121 ± 2	609 ± 14*	738 ± 39 *	700 ± 18 *	113 ± 3	708 ± 33 *	229 ± 6	301 ± 8
	10	0	1	0	64 ± 3	0	987 ± 27 *	834 ± 56 *	282 ± 9
	50	0	0	0	0	0	19 ± 2	0	732 ± 11 *

* MR ≥ 2 compared to control.

**Table 2:** Reverse mutation colonies of four *S. typhimurium* strains exposed to chloroform with and without S9.

Chemicals	Dose (μL/plate)	TA97		TA98		TA100		TA102	
		-S9	+S9	-S9	+S9	-S9	+S9	-S9	+S9
DMSO	100	109 ± 5	95 ± 3	31 ± 1	37 ± 1	185 ± 6	171 ± 6	256 ± 12	254 ± 5
	0	107 ± 7	93 ± 3	33 ± 1	36 ± 2	191 ± 9	165 ± 10	221 ± 11	250 ± 5
chloroform	0.5	93 ± 6	104 ± 4	40 ± 1	42 ± 2	192 ± 9	175 ± 3	244 ± 4	420 ± 14
	2.5	111 ± 6	100 ± 5	35 ± 1	45 ± 3	159 ± 5	175 ± 8	196 ± 16	257 ± 10
	5	100 ± 6	1065 ± 58 *	31 ± 1	32 ± 2	132 ± 9	151 ± 8	224 ± 11	211 ± 8
	10	77 ± 3	1317 ± 39 *	201 ± 6 *	32 ± 1	653 ± 32 *	723 ± 26 *	310 ± 14	1360 ± 29 *
	50	0	0	0	0	0	1	0	2

* MR ≥ 2 compared to control.

**Table 3:** Reverse mutation colonies of four *S. typhimurium* strains exposed to trichloroethylene with and without S9.

Chemicals	Dose (μL/plate)	TA97		TA98		TA100		TA102	
		-S9	+S9	-S9	+S9	-S9	+S9	-S9	+S9
DMSO	100	109 ± 5	95 ± 3	31 ± 1	37 ± 1	185 ± 6	171 ± 6	256 ± 12	254 ± 5
	0	107 ± 7	93 ± 3	33 ± 1	36 ± 2	191 ± 9	165 ± 10	221 ± 11	250 ± 5
trichloro ethylene	0.5	273 ± 14 *	93 ± 4	35 ± 2	309 ± 13 *	147 ± 8	172 ± 8	235 ± 7	329 ± 9
	2.5	820 ± 22 *	896 ± 28 *	38 ± 1	38 ± 0	643 ± 53 *	1664 ± 37 *	281 ± 10	237 ± 8
	5	1000 ± 50 *	449 ± 16 *	920 ± 35 *	32 ± 2	1803 ± 122 *	601 ± 16 *	191 ± 13	269 ± 8
	10	0	41 ± 1	1	36 ± 3	0	501 ± 12 *	611 ± 47 *	249 ± 10
	50	0	36 ± 1	0	32 ± 2	0	296 ± 9	87 ± 6	325 ± 10

* MR ≥ 2 compared to control.

**Table 4:** Reverse mutation colonies of four *S. typhimurium* strains by the mixtures of toluene, chloroform, and trichloroethylene with and without S9.

Chemicals	Dose (μL/plate)	TA97		TA98		TA100		TA102	
		-S9	+S9	-S9	+S9	-S9	+S9	-S9	+S9
DMSO	100	109 ± 5	95 ± 3	31 ± 1	37 ± 1	185 ± 6	171 ± 6	256 ± 12	254 ± 5
	0	107 ± 7	93 ± 3	33 ± 1	36 ± 2	191 ± 9	165 ± 10	221 ± 11	250 ± 5
The mixture	0.1%	111 ± 2	98 ± 1	40 ± 2	41 ± 2	232 ± 6	297 ± 8	277 ± 6	497 ± 4
	1%	92 ± 4	262 ± 3 *	39 ± 5	36 ± 1	225 ± 5	221 ± 6	277 ± 10	287 ± 3
	10%	1	31 ± 3	1	14 ± 2	213 ± 3	1459 ± 47 *	229 ± 6	287 ± 4
	50%	0	0	0	0	213 ± 3	208 ± 7	1320 ± 50 *	210 ± 4
	100%	0	0	0	0	0	1	208 ± 3	1144 ± 29 *

* MR ≥ 2 compared to control.

## Data Availability

The data presented in this study are available on request from the corresponding author.
